# Hypertriglyceridemia Influences the Degree of Postprandial Lipemic Response in Patients with Metabolic Syndrome and Coronary Artery Disease: From the Cordioprev Study

**DOI:** 10.1371/journal.pone.0096297

**Published:** 2014-05-06

**Authors:** Juan F. Alcala-Diaz, Javier Delgado-Lista, Pablo Perez-Martinez, Antonio Garcia-Rios, Carmen Marin, Gracia M. Quintana-Navarro, Purificacion Gomez-Luna, Antonio Camargo, Yolanda Almaden, Javier Caballero, Francisco J. Tinahones, Jose M. Ordovas, Francisco Perez-Jimenez, Jose Lopez-Miranda

**Affiliations:** 1 Unidad de Lípidos y Arteriosclerosis, Departamento de Medicina, Instituto Maimónides de Investigación Biomédica de Córdoba(IMIBIC)/Hospital Universitario Reina Sofía/Universidad de Córdoba, Córdoba, Spain; 2 Centro de Investigación Biomédica en Red de la Fisiopatología de la Obesidad y Nutrición (CIBEROBN), Instituto de Salud Carlos III, Madrid, Spain; 3 Departamento de Análisis Clínicos, Hospital Universitario Reina Sofía, Córdoba, Spain; 4 Hospital Virgen de la Victoria, CIBEROBN, Instituto de Salud Carlos III, Malaga, Spain; 5 Nutrition and Genomics Laboratory, Jean Mayer US Department of Agriculture Human Nutrition Research Center on Aging at Tufts University, Boston, Massachusetts, United States of America; 6 Instituto Madrileño de Estudios Avanzados en Alimentación (IMDEA-Alimentacion), Madrid, Spain; University of Perugia, Italy

## Abstract

**Objective:**

To determine whether metabolic syndrome traits influence the postprandial lipemia response of coronary patients, and whether this influence depends on the number of MetS criteria.

**Materials and Methods:**

1002 coronary artery disease patients from the CORDIOPREV study were submitted to an oral fat load test meal with 0.7 g fat/kg body weight (12% saturated fatty acids, 10% polyunsaturated fatty acids, 43% monounsaturated fatty acids), 10% protein and 25% carbohydrates. Serial blood test analyzing lipid fractions were drawn at 0, 1, 2, 3 and 4 hours during the postprandial state. Total and incremental area under the curves of the different postprandial parameters were calculated following the trapezoid rule to assess the magnitude of change during the postprandial state

**Results:**

Postprandial lipemia response was directly related to the presence of metabolic syndrome. We found a positive association between the number of metabolic syndrome criteria and the response of postprandial plasma triglycerides (p<0.001), area under the curve of triglycerides (p<0.001) and incremental area under the curve of triglycerides (p<0.001). However, the influence of them on postprandial triglycerides remained statistically significant only in those patients without basal hypertriglyceridemia. Interestingly, in stepwise multiple linear regression analysis with the AUC of triglycerides as the dependent variable, only fasting triglycerides, fasting glucose and waist circumference appeared as significant independent (P<0.05) contributors. The multiple lineal regression (R) was 0.77, and fasting triglycerides showed the greatest effect on AUC of triglycerides with a standardized coefficient of 0.75.

**Conclusions:**

Fasting triglycerides are the major contributors to the postprandial triglycerides levels. MetS influences the postprandial response of lipids in patients with coronary heart disease, particularly in non-hypertriglyceridemic patients.

## Introduction

The postprandial state is the period from food intake to post-absorptive state, defined in terms of extent and duration of increased plasma triglycerides (TG) in response to fat intake. It is a dynamic condition, with a continuous fluctuation in the degree of lipemia and glycemia over the day, in which there is a rapid continuous remodeling of the lipoprotein and a host of other metabolic adaptations compared to the relatively stable conditions in the fasting state. Over the last decade, postprandial triglyceride metabolism has taken on more importance, since fasting is not the typical physiological state of humans in modern society, who spend most of the time in the postprandial state. In this context, the evaluation of the postprandial lipemic response may be more important to identify disturbances in lipid metabolism than measurements taken in the fasting state. In fact, large population studies (e.g. Women's Health Study and the Copenhagen City Heart Study) have assessed the association between non-fasting triglycerides and the risk of cardiovascular disease (CVD) events. Data from these studies have clearly documented that postprandial TG levels are excellent markers of risk for coronary artery disease, peripheral vascular disease and cerebrovascular disease [1]–[5]. In this regard, it has been proposed that non-fasting TG (5 mmol/L *vs.* <1 mmol/L) marked a 17- and 5-fold increased risk of myocardial infarction, a 5-and 3-fold increased risk of ischemic stroke, and a 4- and 2-fold increased risk of early death in women and men in the general population [1]–[4].

Moreover, several studies have linked the extent of postprandial lipemia to the incidence of coronary heart disease and it has been proposed that postprandial lipoprotein metabolism is modulated by dietary patterns, food composition, conditions associated with lifestyle (physical activity, smoking and alcohol consumption), physiological factors (age, gender, genetic background and postmenopausal status) and cardiometabolic conditions such as fasting triglycerides levels [6]–[10], type 2 diabetes (T2DM), insulin resistance and obesity [11]–[13].

The importance of Metabolic Syndrome (MetS) lies in its close association with the risk of CVD and T2DM. Unfortunately its prevalence is increasing to epidemic proportions and the health care costs and burden are substantial. One of the most widely accepted definitions is that provided by the National Cholesterol Education Program guidelines, revised in 2004 (rNCEP) [14]. In a recent meta-analysis [15], including 952.083 patients and carried out to assess the prognostic significance of MetS in cardiovascular disease, it was shown that MetS was associated with a 2-fold increase in cardiovascular outcomes (cardiovascular disease, cardiovascular mortality, myocardial infarction and stroke) and a 1.5-fold increase in all-cause mortality. In turn, excluding the influence of the presence of T2DM, this increased risk persists for cardiovascular mortality, acute myocardial infarction and stroke. These data confirmed previously published evidence [16]. From a clinical point of view, there is a debate as to whether the MetS alone or its associated conditions are more important for CVD incidence and mortality or whether prevention and/or treatment of the MetS will reduce CVD incidence and mortality. In this regard, previous observations have reported that the presence of more components of MetS was associated with an increase in subclinical atherosclerosis, and incidence and mortality of coronary heart disease [17]–[21]. In the same context, it has been suggested that, in healthy people, there is a relationship between MetS components and exacerbated postprandial lipemia [22], but there is still a lack of data in patients with CVD.

Based on this previous evidence, our objective was to determine if MetS traits influence the postprandial lipemia of coronary patients, and whether this influence depends on the number of MetS criteria.

## Materials and Methods

### Ethics Statement

Patients gave written informed consent to participate in the study. The trial protocol and all amendments were approved by the Ethics Committee from Reina Sofia University Hospital, following the Declaration of Helsinki (2008) of the World Medical Association.

### Population

The current work was conducted within the framework of the CORDIOPREV study. The CORDIOPREV study is an ongoing prospective, randomized, opened, controlled trial including 1002 patients with coronary heart disease (CHD), who had their last coronary event more than six months before enrollment in two different dietary models (Mediterranean and low-fat) over a period of five years, in addition to conventional treatment for CHD.

Patients were recruited from November 2009 to February 2012, mostly at the Reina Sofia University Hospital (Cordoba, Spain), but patients from other hospital centers from the Cordoba and Jaen provinces were also admitted.

Inclusion and exclusion criteria are shown in **Table 1**. In summary, patients were eligible if they were between 20 and 75, had established CHD without clinical events in the last six months, were thought to follow a long-term dietary intervention and had no severe diseases or an expected life expectancy of under five years. Patients were categorized depending on the presence or not of MetS and number of its criteria, defined by the rNCEP criteria [14].

**Table 1:** Inclusion and exclusion criteria for the CORDIOPREV study.

Ages Eligible for Study	20 to 75
Genders Eligible for Study	Both
**Inclusion Criteria**	Unstable Coronary Disease Chronic
	Acute Myocardial Infarction
	Unstable Angina
	Chronic Coronary Disease with high risk of event
**Exclusion Criteria**	Age <20 or >75 (or life expectancy below 5 years)
	Patients already scheduled for revascularization Patients submitted to revascularization in the last 6 months Grade II/IV Heart failure
	Left ventricle dysfunction with ejection fraction lower than 35%
	Patients unable to follow a protocol
	Patients with severe uncontrolled Diabetes Mellitus, or those with Renal Insufficiency with permanent plasma creatinine higher than 2 mg/dl, or cerebral complications of Diabetes Mellitus
	Other chronic diseases: Psychiatric diseases, Chronic Renal Insufficiency, Chronic Hepatopathy, Active Malignancy, Chronic Obstructive Pulmonary Disease, Diseases of the digestive tract, Endocrine disorders, Patients participating in other clinical trials (at the time of enrollment or 30 days before)

### Study design

Before participants were enrolled in the two different dietary models from CORDIOPREV study, they received an oral fat tolerance test using a weight-adjusted meal (0.7 g fat and 5 mg cholesterol per kg body weight) with 12% saturated fatty acids (SFA), 10% polyunsaturated fatty acids (PUFA), 43% monounsaturated fatty acids (MUFA), 10% protein and 25% carbohydrates (CHO). The meal composition was designed by a group of nutritionists with olive oil, skimmed milk, white bread, cooked egg yolks and tomatoes.

### Methodology of the oral fat tolerance test

Before starting the test, the patients had fasted (food/drugs) for 12 hours and were asked to refrain from smoking during the fasting period and from alcohol intake during the preceding 7 days. They were also asked to avoid strenuous physical activity the day before the test was given. The patients arrived at the clinical center at 08:00 h. We measured anthropometric (weight, height, waist circumference, Body mass index (BMI) and blood pressure) and biochemical measurements, took a fasting blood sample and under supervision, the patients ingested the fatty food meal. The breakfast was eaten in 20 min. After the meal, the volunteers rested and consumed no food for 5 hours, but were allowed to drink water.

Blood samples for biochemical testing were collected before the meal and every hour during the next 4 hours, following recommendations for an oral fat tolerance test proposed by Mihas et al. in a recent meta-analysis [23].

### Laboratory test

Venous blood was sampled from the antecubital vein and collected into tubes with no anticoagulant and EDTA, and immediately transferred to 4°C. To minimize proteolytic degradation, plasma was supplemented with protease inhibitor cocktail 40 µL per mL of plasma. Plasma and serum samples were frozen at −80°C for further biochemical analysis.

Serum parameters were measured using spectrophotometric techniques (enzymatic colorimetric methods): hexokinase method for glucose, and oxidation-peroxidation for total cholesterol, HDL-cholesterol and triglycerides. The LDL-cholesterol was calculated using the Friedewald formula (provided the triglyceride level was less than 300 mg/dl). Apolipoprotein A1 and apolipoprotein B were determined by immunoturbidimetry by means of mouse specific antibodies for every magnitude.

TG-rich lipoprotein fraction (TRL) containing chylomicrons and VLDL was removed from plasma by ultracentrifugation performed in a 70Ti fixed-angle rotor at 30.000 rpm and 4°C for 30 min. at d<1.006 g/mL.

### Statistical

All statistical analyses were made with PASW Statistics software, version 18.0.0. Continuous variables were compared using Student's “t” and the analysis of variance (ANOVA) depending on the existence of two or more groups in each comparison. When these variables did not follow a normal distribution, the required transformation of the data was used for analysis. We used total (AUC) and incremental (iAUC) area under the curves of the different postprandial parameters following the trapezoid rule to assess the magnitude of change during the postprandial state, as in previous works by our group [24]. The units used for the AUC and iAUC were (mg * min * dL^−1^). To determine the influence of metabolic syndrome in the postprandial metabolism, we used a general linear model of repeated measures of each postprandial parameter, with presence or not of metabolic syndrome as a between-subjects variable, blood drawing time as a within-subject variable and gender and age as covariates. Bonferroni's correction was used for multiple comparisons. Pearson's correlation or Spearman rank order correlation analyses were performed to examine the correlations between the levels of metabolic syndrome traits (Systolic blood pressure, Diastolic Blood Pressure, HDL-c, TG, Glucose and waist circumference), treatment (statins and fibrates) and AUC of postprandial parameters. The values of fasting triglycerides, fasting glucose, fasting HDL-c, waist circumference and both systolic and diastolic blood pressure were tested in a stepwise multiple linear regression to predict the AUC of triglycerides and determine their individual effect on it.

## Results

### Baseline characteristics

**Figure 1:**
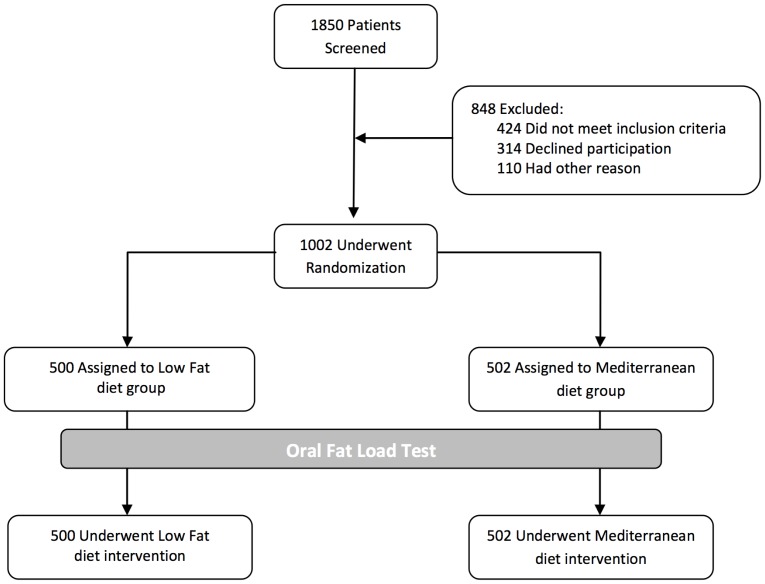
Flow-chart of CORDIOPREV study. Before participants were enrolled in the two different dietary models from CORDIOPREV study, they received an oral fat tolerance test using a weight-adjusted meal (0.7 g fat and 5 mg cholesterol per kg body weight) with 12% saturated fatty acids (SFA), 10% polyunsaturated fatty acids (PUFA), 43% monounsaturated fatty acids (MUFA), 10% protein and 25% carbohydrates (CHO).

A total of 1002 participants with coronary artery disease were included in the CORDIOPREV study (**Figure 1**), of which 581 had MetS criteria.

**Table 2** shows the baseline characteristics. The mean age was 59.5 years for all the population. They were mostly males (83.4%) with a mean body mass index of 31.1 Kg/m^2^. Patients with MetS showed significant differences compared with patients without MetS, with greater weight, waist circumference, body mass index, plasma glucose, and higher levels of fasting TG and ApoB (all, p<0.05). MetS patients showed lower levels of fasting HDL-c, ApoA1 and LDL-c (all, p<0.05).

**Table 2:** Baseline characteristics of the patients.

	All patients	Metabolic Syndrome	Non-Metabolic Syndrome	p-value
	(n = 1002)	(n = 581)	(n = 421)	
**Age (Years)**	59.5±0.2	60.0±0.3	58.9±0.4	NS
**Male/Female**	837/165	470/111	367/54	<0.001
**Weight (Kg)**	85.1±0.4	88.8±0.6	80.1±0.6	<0.001
**Waist circumference**	105.1±0.3	108.7±0.4	100.1±0.5	<0.001
**BMI (kg/m2)**	31.1±0.1	32.4±0.1	29.3±0.2	<0.001
**HDL-c (mg/dL)**	42.2±0.3	38.6±0.4	47.1±0.5	<0.001
**Fasting Plasma Glucose (mg/dL)**	113.7±1.2	125.9±1.8	97.1±1.0	<0.001
**TG (mg/dL)**	135.4±2.2	159.9±3.1	102.1±2.2	<0.001
**APO-A1 (mg/dL)**	129.6±0.7	124.9±0.8	136.1±1.1	<0.001
**Total Cholesterol (mg/dL)**	159.0±0.9	158.6±1.3	159.5±1.4	NS
**APO-B (mg/dL)**	73.6±0.5	76.1±0.8	70.2±0.8	<0.001
**LDL-c (mg/dL)**	88.5±0.8	86.37±1.1	91.5±1.2	<0.001
**Lipid lowering drugs:**				
** Statins (%)**	85.6	85.7	85.5	NS
** Fibrates (%)**	1.6	2.4	0.5	0.01
** Other** 1 **(%)**	4.8	4.6	4.9	NS

Values are means ±SEM. Continuous variables were compared using the analysis of variance (ANOVA). Qualitative variables were compared using Chi Square test. BMI = Body mass index. HDL-c = High density lipoprotein cholesterol. TG = Triglycerides. LDL-c = Low density lipoprotein.

1 Other lipid lowering drugs: Ezetimibe and Nicotinic acid.

### Postprandial parameters and Metabolic Syndrome

**Figure 2:**
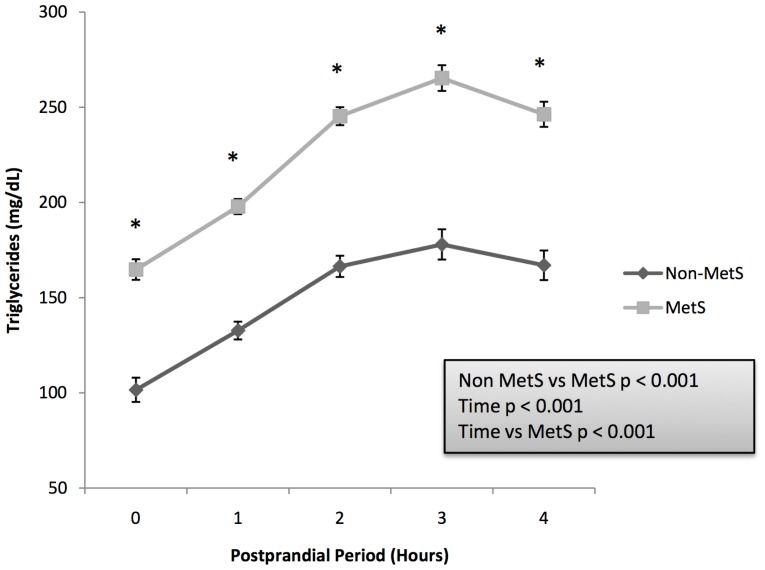
Plasma levels of triglycerides (mg/dL) during postprandial period in patients with and without MetS. MetS patients showed higher plasma levels of triglycerides in each of the time points performed during the postprandial period (p<0.001). Results are plotted as Mean±SE. Variables were compared using repeated measured ANOVA, with sex and age as covariates.

After the intake of the fat load test, we found differences between patients with and without MetS during the postprandial period. MetS patients showed higher plasma levels of TG (**Figure 2**) and ApoB as well as lower HDL-c and ApoA1 in each of the time points performed during the postprandial period (all, p≤0.001). No differences were detected for total cholesterol between groups. Furthermore, MetS patients showed lower AUC of postprandial HDL-c (8375.6±84.6 vs 9940.5±99.8, p<0.001) and ApoA1 (28236.1±205.7 vs 30546.9±242.9, p<0.001), with higher AUC of postprandial TG (55048.74±1186.7 vs 36373. 6±1398.3, p<0.001), TRL (22389.3±606.9 vs 13983.4±703.1, p<0.001), ApoB (16697.4±176.8 vs 15289.7±207.7, p<0.001) and glucose (36936.7±568.4 vs 27546.3±669.5, p<0.001). We also analyzed the incremental (iAUC) of area under postprandial parameters curve. MetS patients showed higher iAUC of TG (15298.9±540.1 vs 12296.2±636.4, p<0.001), TRL (12673.1±353.6 vs 9322.5±409.6) and glucose (5608.0±286.7 vs 2131.9±337.6, p<0.001), with higher negative iAUC of ApoB (−743.2±68.4 vs −490.2±80.3, p<0.001) and total cholesterol (−1143.3±92.9 vs −828.5±109.8, p<0.001).

### Components of Metabolic Syndrome and postprandial response

**Figure 3:**
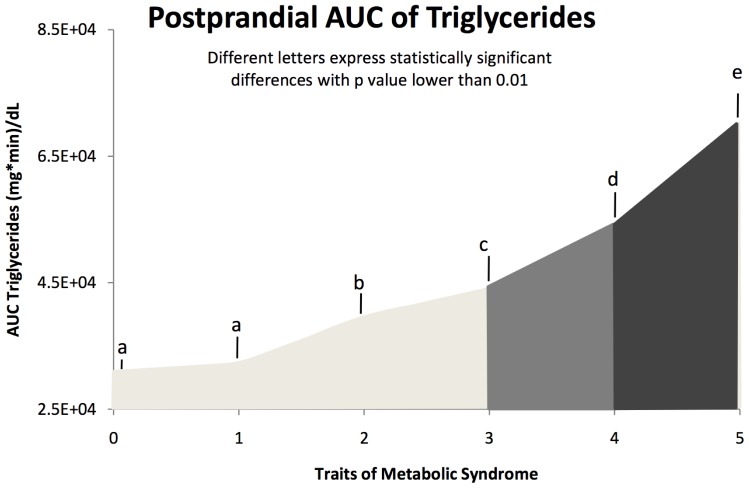
Postprandial AUC of triglycerides in relation to Mets traits. The magnitude of the AUC of postprandial TG increased in the sequence 0, 1<2 criteria <3 criteria <4 criteria <5 criteria. Variables were compared using ANOVA with sex and age as covariates. Different letters express statistically significant differences with a p value below 0.01.

Postprandial lipemia response was directly related to the number of MetS components. Specifically, we found a positive association between the number of MetS criteria and the response of postprandial plasma TG (p<0.001), AUC of TG (p<0.001) and iAUC of TG (0<0.001). Interestingly, the magnitude of the AUC of TG increased in the sequence 0, 1<2 criteria <3 criteria <4 criteria <5 criteria, as shown in **Figure 3** (p<0.001). For the iAUC of TG, this sequence differs as follows: 0, 1<2, 3<4<5 (10179±1862, 10501±828<13746±641, 13774±633<15886±653<19006±1070, respectively). A similar fashion was detected for AUC of plasma glucose and ApoB. In contrast, a negative relationship in the number of criteria of MetS with AUC of HDL-c and ApoA1 was observed (data not shown).

The correlations between the levels of MetS traits and postprandial parameters (AUCs of TG, TRL, total cholesterol, HDL-c, ApoA1, ApoB and glucose) are shown in **Table 3**. Specifically, postprandial AUC of TG were significantly correlated with the values of diastolic blood pressure (r = 0.11, p<0.01), HDL-c (r = −0.27, p<0.01), fasting glucose (r = 0.20, p<0.01), TG (r = 0.77, p<0.01) and waist circumference (r = 0.20, p<0.01). Moreover, levels of postprandial AUC of TRL were significantly correlated with the values of diastolic blood pressure (r = −0.13, p<0.01), HDL-c (r = −0.25, p<0.01), TG (r = 0.58, p<0.01), fasting glucose (r = 0.16, p<0.01) and waist circumference(r = 0.15, p<0.01). Hypolipidemic drugs (statins or fibrates) were not significantly correlated with fasting TG and the AUC of TG (all p>0.05).

**Table 3:** Correlations between Mets traits and area under the curve of postprandial parameters.

	Triglycerides	TRL-TG	TC	HDL-c	Apo-A1	Apo-B	Glucose
	r^1^						
	(p values)						
**SBP, mmHg**	0.03	0.01	0.07	−0.01	0.02	0.09	0.17
	(0.30)	(0.67)	(0.03)	(0.96)	(0.40)	(<0.01)	(<0.01)
**DBP, mmHg**	0.11	0.13	0.13	0.01	0.01	0.14	−0.02
	(<0.01)	(<0.01)	(<0.01)	(0.92)	(0.58)	(<0.01)	(0.52)
**HDL-C, mg/dL**	−0.27	−0.25	0.24	0.82	0.68	−0.02	−0.18
	(<0.01)	(<0.01)	(<0.01)	(<0.01)	(<0.01)	(0.47)	(<0.01)
**TG, mg/dL**	0.77	0.58	0.30	−0.31	−0.14	0.39	0.17
	(<0.01)	(<0.01)	(<0.01)	(<0.01)	(<0.01)	(<0.01)	(<0.01)
**Glucose, mg/dL**	0.20	0.16	0.02	−0.12	−0.09	0.06	0.80
	(<0.01)	(<0.01)	(0.44)	(<0.01)	(<0.01)	(0.04)	(<0.01)
**Waist circumference, cm**	0.20	0.15	−0.01	−0.15	−0.10	0.03	0.24
	(<0.01)	(<0.01)	(0.87)	(<0.01)	(<0.01)	(0.31)	(<0.01)

1 Values are shown as correlation coefficients and (p values). DBP, diastolic blood pressure; HDL-C, high density lipoprotein-cholesterol; SBP, systolic blood pressure; TC, Total cholesterol; TG, triglycerides.

In stepwise multiple linear regression analysis with the AUC of triglycerides as the dependent variable, only fasting triglycerides, fasting glucose and waist circumference appeared as significant (P<0.05) contributors. The multiple regression (R) was 0.77, and fasting triglycerides showed the greatest effect on AUC of triglyceride (**Table 4**).

**Table 4:** Multiple linear regression coefficients^1^ to predict AUC of triglycerides.

Parameter	Unstandardized Coefficients		Standardized Coefficients	Sig.
	B	Std. Error	Beta	
**Fasting TG**	228.49	6.68	0.752	<0.001
**Fasting Glucose**	29.41	11.05	0.059	0.008
**Waist Circumference**	85.78	37.43	0.051	0.02

Predictive variables tested by stepwise method: fasting triglycerides (mg/dL), fasting glucose (mg/dL), waist circumference (cm), fasting HDL-c (mg/dL), systolic and diastolic Blood (mmHg).

1 (Constant) = 2163.28. (R^2^) = 0.602.

To explore the effect of basal hypertriglyceridemia on postprandial metabolism, patients were divided into two groups according to the presence or abscence of basal hypertriglyceridemia. In patients with high fasting triglycerides (TG ≥150 mg/dL), the AUC and iAUC of TG were significantly greater (64164±1169 vs 36403±501, p<0.001; and 17548±1083 vs 12229±324, p = 0.001, respectively) than in the group of patients with fasting TG<150 mg/dL.

The influence of the different MetS factors still remained statistically significant (p<0.001) when we analyzed the AUC of TG on those patients without high TG at the basal point, but not on those patients with basal hypertriglyceridemia (**Table 5**).

**Table 5:** Area under the curve of postprandial triglycerides in patients with and without basal hypertriglyceridemia in relation to Metabolic Syndrome components.

TG<150 mg/dL							
	Traits of Metabolic Syndrome						
	0	1	2	3	4	5	global p value
**AUC TG**	31249.9^3,4^	32162.0^2,3,4^	36180.3^1,4^	37815.1^0,1,4^	41472.0^0,1,2,3^	-	1.01×10^−7^
	(2364.3)	(1060.3)	(881.2)	(920.7)	(1219.2)		

TG≥150 mg/dL							
	Traits of Metabolic Syndrome						
	0	1	2	3	4	5	global p value
**AUC TG**	-	63198.2	58217.3^5^	62995.1	64265.9	67544.7^2^	NS
		(19253.4)	(3362.4)	(2607.4)	(1852.1)	(2300.4)	

Area under the curve of triglycerides is expressed as mg*min*dL^−1^. Values are shown as Mean (SEM) and were compared using ANOVA and Bonferroni multiple comparison post hoc test with sex and age as covariates. Superscript characters indicate differences between groups (0, 1, 2, 3, 4, 5) within the same row.

## Discussion

In the present study we investigated the effect of a fatty meal on postprandial lipid metabolism in patients with coronary artery disease. We showed that MetS and the number of its components influence the degree of postprandial lipemic response. Specifically, postprandial AUC of TG showed a progressively unfavorable increase from one component to five in our population. However, this effect was attenuated when the population was divided into two groups according to the presence or absence of basal hypertriglyceridemia. Thus, only those patients without high fasting TG remained a statistically significant influence.

Recently, it has been established that the presence of higher number of components of MetS is associated with an increase in subclinical atherosclerosis, and incidence and mortality of CHD. Teramura et al. reported that intima-medial thickness was significantly higher in subjects with MetS and increased with the number of coexisting components of MetS, compared with those without MetS [17]. Furthermore, a prospective cohort study including 6255 subjects, showed how CHD and CVD mortality were both influenced by the number of MetS components [20]. In the same context, Sattar et al. observed that men presenting four or five MetS traits had a 3.7-fold increase in risk for CHD and a 24.5-fold increase for diabetes compared with men with none [19]. However, the mechanisms underlying this fact are still unknown. Although it is generally accepted that the main pathogenic mechanism underlying the development of cardiometabolic changes in patients with MetS relies on insulin resistance, other mechanisms could influence the increased risk of CVD associated to MetS. While the independence of the association and causality has not been fully established, postprandial TG concentrations have emerged as a clinically significant CVD risk factor following the results of several prospective studies [25]. In our study, patients with an increased number of MetS components showed higher levels of postprandial TG, confirmed by AUC and iAUC of TG. This deterioration in postprandial lipid metabolism associated with the increase number of MetS components may favor a higher risk of atherogenesis.

Previous studies have explored the mechanisms underlying the relation between postprandial lipid metabolism and the increased risk of atherogenesis [26], [27]. High levels of postprandial triglycerides have been reported to correlate with high remnant cholesterol in individuals in the general population [4], and, in addition, it has been proposed that in those situations where the liver induces an overproduction of VLDL, such as central obesity, metabolic syndrome, type 2 diabetes mellitus and familial combined hypercholesterolemia, VLDL and chylomicrons catabolic mechanisms are saturated [12], [28]–[30]. These mechanisms cause the accumulation of VLDL and chylomicron remnants [31]–[33], a lower concentration of HDL-c and the activation of leukocytes and endothelial cells by the remnants and fatty acids [34], [35]. At this stage, postprandial remnant lipoproteins would penetrate the vessel wall and monocytes would catch them, inducing the formation of foam cells [36]. Although it is generally accepted that the main pathogenic mechanism underlying the development of metabolic changes in patients with MetS relies on insulin resistance, a large body of evidence supports the concept that increased oxidative stress and a state of chronic low-level inflammation may have important roles in MetS-related manifestations [37]. In this way, the formation of oxidized reactive species and oxidized remnant lipoproteins would also contribute to endothelial dysfunction and the development of coronary artery disease [37]. In this regard, we have recently demonstrated the relationship between the number of MetS components and the degree of oxidative stress in MetS patients [38].

Previous evidence carried out in healthy population has suggested a significant linear trend between increasing numbers of MetS components and magnitude of postprandial lipemia in 112 healthy subjects [25]. Nevertheless, to our knowledge, our study is the first one to show that in non-hypertriglyceridemic coronary patients. Another important feature underlying MetS is atherogenic dyslipidemia, defined as a rise in triglycerides and small LDL particles and low HDL-c [39]. In this regard, we have observed that patients with at least three MetS components have higher ApoB plasma levels and lower HDL-c and ApoA1 plasma levels in all blood drawn during the postprandial state, as well as a positive relationship with AUC of ApoB and a negative relationship with AUC of HDL-c and ApoA1. All of these abnormalities have been implicated as being independently atherogenic [14].

Although baseline TG has been previously proposed in different studies as the major determinant of postprandial lipemia [7]–[10], in our study the involvement of other factors were also statistically significant. Thereby, the stepwise multiple linear regression analysis with the AUC of triglycerides as the dependent variable, showed that fasting TG, fasting glucose and waist circumference appeared as significant independent contributors, with fasting TG as the major contributors (see Table 4).To avoid the influence of high levels of fasting TG on postprandial response, patients were divided in our study into two groups on the basis of their fasting TG concentrations. In hypertriglyceridemic patients, the AUC and iAUC of TG were significantly greater than in the group of normotriglyceridemic patients, according to previous data reported [40]. Besides, the influence of number of MetS components on AUC of TG remained statistically significant in those patients without high fasting TG but not in those patients with basal hypertriglyceridemia. This feature may be related to the fact that in an already disturbed background, as suggested by a fasting hypertriglyceridemia, the postprandial lipid metabolism is altered, and cannot be impaired further by the presence of MetS traits. However, in patients that are not hypertriglyceridemic, the addition of different metabolic syndrome criteria can progressively worsen the efficient management of a fat meal, suggesting, from a clinical point of view, that MetS subjects with normotriglyceridemia would obtain a higher benefit on the size of postprandial lipemia controlling MetS components than those with hypertriglyceridemia.

Despite the great strength of our study given the population size and the standardized methodology used, there were some limitations. The cross-sectional study design limited our ability to make an inference about the casual relationship between MetS components and postprandial parameters. However, it will be possible to evaluate this point in the future taking in consideration that the CORDIOPREV study is an ongoing prospective, randomized, opened and controlled trial with a mean proposed follow-up of 5 years. Moreover, it would be interesting to study whether those patients with an increased number of MetS components and higher postprandial lipemia have more cardiovascular events in the future.

In summary, our study shows that the existence of MetS influences the postprandial response of carbohydrates and lipids in patients with coronary heart disease. In non-hypertriglyceridemics patients, the magnitude of postprandial response is related to the number of MetS components altered. Fasting triglycerides are the major contributors to the postprandial triglycerides levels. Our findings imply the need for intensive control of MetS components to decrease the cardiovascular risk.
